# Provider Visual Attention Correlates With the Quality of Pediatric Resuscitation: An Observational Eye-Tracking Study

**DOI:** 10.3389/fped.2022.867304

**Published:** 2022-05-24

**Authors:** Peter Gröpel, Michael Wagner, Katharina Bibl, Hannah Schwarz, Felix Eibensteiner, Angelika Berger, Francesco S. Cardona

**Affiliations:** ^1^Division of Sport Psychology, Department of Sport Sciences, Centre for Sport Science and University Sports, University of Vienna, Vienna, Austria; ^2^Division of Neonatology, Pediatric Intensive Care and Neuropediatrics, Department of Pediatrics, Comprehensive Center for Pediatrics, Medical University of Vienna, Vienna, Austria; ^3^Department of Emergency Medicine, Medical University of Vienna, Vienna, Austria

**Keywords:** resuscitation, basic life support (BLS), attention, gaze, eye-tracking (ET), performance, simulation

## Abstract

**Background:**

Eye-tracking devices are an innovative tool to understand providers’ attention during stressful medical tasks. The knowledge about what gaze behaviors improve (or harm) the quality of clinical care can substantially improve medical training. The aim of this study is to identify gaze behaviors that are related to the quality of pediatric resuscitation.

**Methods:**

Forty students and healthcare providers performed a simulated pediatric life support scenario, consisting of a chest compression task and a ventilation task, while wearing eye-tracking glasses. Skill Reporter software measured chest compression (CC) quality and Neo Training software measured ventilation quality. Main eye-tracking parameters were ratio [the number of participants who attended a certain area of interest (AOI)], dwell time (total amount of time a participant attended an AOI), the number of revisits (how often a participant returned his gaze to an AOI), and the number of transitions between AOIs.

**Results:**

The most salient AOIs were infant chest and ventilation mask (ratio = 100%). During CC task, 41% of participants also focused on ventilation bag and 59% on study nurse. During ventilation task, the ratio was 61% for ventilation bag and 36% for study nurse. Percentage of correct CC rate was positively correlated with dwell time on infant chest (*p* = 0.044), while the overall CC quality was negatively correlated with dwelling outside of pre-defined task-relevant AOIs (*p* = 0.018). Furthermore, more dwell time on infant chest predicted lower leakage (*p* = 0.042). The number of transitions between AOIs was unrelated to CC parameters, but correlated negatively with mask leak during ventilations (*p* = 0.014). Participants with high leakage shifted their gaze more often between ventilation bag, ventilation mask, and task-irrelevant environment.

**Conclusion:**

Infant chest and ventilation mask are the most salient AOIs in pediatric basic life support. Especially the infant chest AOI gives beneficial information for the resuscitation provider. In contrast, attention to task-irrelevant environment and frequent gaze shifts seem to harm the quality of care.

## Introduction

Pediatric cardiac arrest is a rare yet highly stressful event (1, 2). Resuscitation providers must work under high cognitive load and time pressure, which often leads to errors and deviations from proper protocol (3). To address this issue, simulation-based programs have been tested and recommended for medical education (4, 5). These programs effectively improved resuscitators’ technical skills, but deficiencies in attentional and communication skills remained common and responsible for the majority of fatal errors and poor patient outcomes (6, 7). Consequently, it is imperative that healthcare providers (HCPs) are not only experienced in emergency procedures, but are also able to stay focused and work efficiently under pressure.

This study addresses attentional mechanisms related to the quality of resuscitation. Psychological research has shown that pressure causes attention to shift from task-relevant to irrelevant cues, which may result in disregarding important information and impaired performance (8). Yet the performance-harming effect of pressure is not inevitable; performers who have incorporated task-relevant cues into their execution routines perform better under pressure (9). Consequently, the knowledge about what are the most important task-relevant cues (and gaze behaviors) in pediatric resuscitation may substantially improve medical education and in turn the quality of clinical care.

Eye-tracking devices are an innovative tool to understand HCPs’ attention during stressful medical tasks (10). Evidence on visual attention during neonatal and pediatric resuscitation shows that resuscitators mostly focus on the infant, displays, and airway equipment (11–15). Experts pay more attention to the patient’s chest and airway than non-experts (16). These results imply that focusing on the patient and airway equipment are the most important gaze behaviors in pediatric resuscitation. However, evidence whether or not these gaze behaviors directly predict the quality of care is still missing from the literature.

The aim of this study is to identify HCPs’ gaze behaviors that are related to the quality of pediatric resuscitation. This may help to advance medical training and propose relevant educational procedures. Based on the above findings, the infant and airway equipment represent the most salient cues or areas of interests (AOIs) in pediatric resuscitation. We thus hypothesize that the infant and airway equipment AOIs will be fixated by our participants and revisited more often than any other AOIs in a simulated resuscitation scenario. We further hypothesize that dwelling and refocusing on the infant and airway equipment AOIs is positively related to the quality of care.

## Materials and Methods

### Participants and Study Design

This study was a secondary quantitative analysis of a randomized cross-over simulation study carried out in 2020, which examined the effect of feedback devices on visual attention and the quality of pediatric resuscitation (17). Originally, participants completed two pairs of scenarios: a chest compression (CC) scenario with and without a visible feedback device, followed by a ventilation scenario again with and without feedback. The feedback device was either visible or hidden from the participant, but always recording. It is important to note that we only included the conditions without any visible feedback device in this secondary analysis. This was because we did not want to bias the effect of gaze behavior on the quality of resuscitation by having an onsite feedback device. The presence of the feedback device caused participants to shift their attention to that device to a large extent, thereby substantially reducing attention to other stimuli (17). Consequently, the actual effect of gaze behavior on the quality of resuscitation could only be analyzed in the conditions without the feedback device. The present secondary analysis had an observational study character.

The original study was conducted at the Pediatric Simulation Training Center at the Medical University of Vienna, Austria. The study protocol was reviewed according to the Consolidated Standards of Reporting Trials approach, with the extension for simulation-based research (18). The Ethics Committee of the Medical University of Vienna gave this study an exempt status. Participants were medical students in their final year, fellows, nurses, and consultants from the Division of Neonatology, Pediatric Intensive Care and Neuropediatrics. Inclusion criteria were: medical students or HCPs affiliated with the Medical University of Vienna; available for 30 min; and provision of informed consent. Exclusion criterium was participation in previous eye-tracking simulation studies at our Simulation Training Center. All participants signed informed consent prior to participation and were then randomly assigned to perform CC twice (with and without feedback), and ventilations twice afterward. Hence, each participant completed a total of four basic life support (BLS) scenarios according to the European Resuscitation Council (ERC) pediatric BLS guidelines (15:2) in a cross-over setting (19). The visibility of the feedback device was randomized (sealed envelope) within each pair of scenarios. Participants were wearing head-mounted eye-tracking glasses (Tobii Pro 2.0; Tobii AB, Danderyd, Sweden) to record their gaze behavior during all scenarios.

### Study Procedure, Equipment, and Parameters

At the start of the study, participants were briefed on the study, received a brief review of the current pediatric resuscitation guidelines, and completed a questionnaire on demographic data and expertise in pediatric resuscitation. Thereafter, the eye-tracking glasses were calibrated according to the company’s instructions by fixating a standardized calibration card with a black circle and a black dot in the middle at a distance of one meter. Calibration was performed before each scenario and had to be approved by the recording software before measurement. Participants then completed the four BLS scenarios. The resuscitation team consisted of the participant and a study nurse. Each scenario lasted for 3 min and all tasks were done in one session.

The QCPR Baby manikin (Laerdal Medical, Stavanger, Norway) was used in the CC scenarios, whereas the SimNewB manikin (Laerdal Medical, Stavanger, Norway) was used in the ventilation scenarios because it has no internal air leak. For the quality of CCs, the QCPR Baby was connected to the SimPad Plus Skill Reporter software (Laerdal, Stavanger, Norway) which recorded CC rate, CC rate compliance (percentage of correct CC rate), depth, depth compliance (percentage of correct CC depth), complete release, and hand position. In addition, the total compression quality score (%), which is the composite score of the above parameters, was calculated and provided by the software. Participants received 100% if the guideline criteria for each variable (CC rate of 100–120 CCs per minute; depth of 4 cm; complete chest recoil between each CC; optimal hand position) were executed accurately (19). For the quality of ventilations, a flow sensor (Neo Training, Monivent AB, Gothenburg, Sweden) was placed between the face mask (CareFusion Vital Signs Infant Face Mask, Châteaubriant, France) and the bag (Laerdal Silicone Resuscitator Pediatric Basic, Stavanger, Norway) to measure inspiratory (V_*Ti*_) and expiratory tidal volume (V_*Te*_), peak inspiratory pressure, and mask leak. Inspiratory pressure of <30 cmH_2_O, tidal volumes between 4–8 mL/kg, and low leakage (as low as possible) reflect high quality of ventilation (1).

**FIGURE 1:**
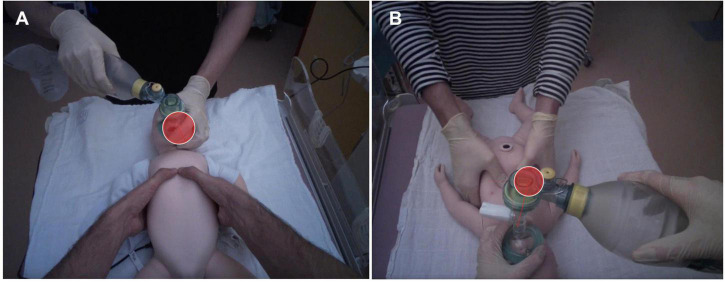
Snapshots showing visual attention (red circle) of participants during the chest compression scenario **(A)** and the ventilation scenario **(B)**.

The eye-tracking glasses recorded a first-person view video with an overlying pupil fixation showing where participants were looking in real time (Figure 1). Eye movements were sampled at a rate of 50 Hz and analyzed with the Tobii Pro Glasses Analyzer software (Tobii AB, Danderyd, Sweden). Clinical experts of the research team determined six areas of interest (AOIs) before the study: (1) feedback device (if available), (2) infant chest, (3) ventilation mask, (4) ventilation bag, (5) study nurse, and (6) others (i.e., focusing outside of the predefined AOIs). Because we only analyzed CC and ventilation scenarios without a visible feedback device in this secondary analysis, the feedback device AOI was not relevant. The analyzed eye-tracking parameters were ratio (the proportion of participants who fixated a particular AOI), dwell time (total amount of time a participant attended an AOI), the number of revisits (how often a participant returned his gaze to an AOI), and the number of transitions between AOIs. Higher ratio and higher number of revisits both reflect the relative importance of an AOI, longer dwell time on an AOI represents conscious attention paid to that AOI, while high number of transitions between AOIs indicates insecurity or (inefficient) search in multiple information sources (20).

### Statistical Analysis

Descriptive statistics were used to describe the sample and the ratio of each AOI. Repeated measures analysis of variance (ANOVA) with Bonferroni corrected *post hoc* comparisons were used to test the differences in revisits among the AOIs. Partial correlations were used to depict the relationship between visual attention, chest compression, and ventilation parameters, with controlling for the order effect (no-feedback condition first vs. second). Recall that, in the original study, participants performed both CC and ventilation tasks twice (with and without the feedback device), while being randomized whether they start with the feedback or the non-feedback condition. Participants who started with the feedback condition performed in the subsequent non-feedback condition significantly better than participants who directly started with the non-feedback condition (*p* < 0.05). Because only the non-feedback condition was analyzed in this study, we included the order of the non-feedback condition as a covariate in the above analyses. All above analyses were performed with SPSS 27.0 (IBM Corp., Armonk, NY, United States). The level of significance was set at *p* < 0.05 (two-tailed). Parameters with a skewed data distribution were log transformed before analysis. Sample size calculation was conducted for the original study which employed a randomized cross-over design (17), and was therefore irrelevant for the observational design of this secondary analysis. However, sensitivity analysis with the G*Power software determined that partial correlations of 0.21 and higher could be reliably detected with our sample size by the alpha level of 0.05 and power of 0.80.

## Results

We collected data from 40 participants (25 females and 15 males) who were either medical students (*n* = 9), fellows (*n* = 22), consultants (*n* = 8), or a nurse (*n* = 1). Their clinical experience ranged from 0 to 26 years (*M* = 4.26, *SD* = 6.52). The majority of participants (98%) had prior experience in simulation-based resuscitation training and felt competent in providing BLS (92%).

### Visual Attention and Chest Compression Quality

**FIGURE 2:**
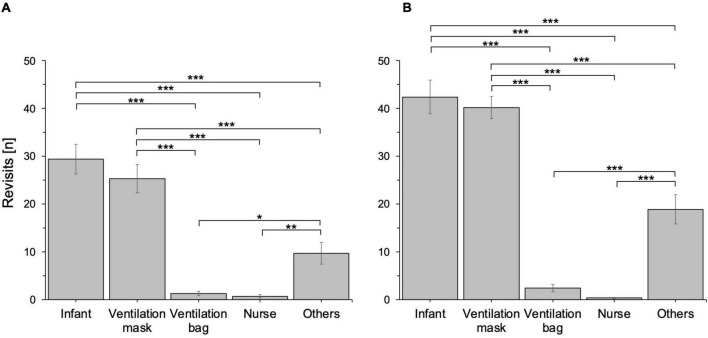
Mean number of revisits to the study AOIs during the chest compression scenario **(A)** and the ventilation scenario **(B)**. Error bars are standard errors of the mean (SEM). *N* = 40, *p < 0.05, **p < 0.01, ***p < 0.001.

Table 1 shows descriptive statistics of the tested eye-tracking parameters. The most salient AOIs were infant chest and ventilation mask (both ratios = 100%), followed by study nurse (59%) and ventilation bag (41%). These results were mirrored by significant differences in revisits among the AOIs (*F* = 51.19, *p* < 0.001), with participants returning their gaze to infant chest and ventilation mask more often than to any other AOI (*p* < 0.001; Figure 2). However, attention outside of task-relevant stimuli (the “others” AOI) was also common (ratio = 97%) and more frequent than attention paid to the study nurse (*p* = 0.004) and ventilation bag AOIs (*p* = 0.011).

**TABLE 1:** Descriptive statistics of the tested eye-tracking parameters.

	Ratio	Dwell time (s)		Revisits (n)		Transitions (n)	
	%	*Mean*	*SD*	*Mean*	*SD*	*Mean*	*SD*
**AOI (CC task)**						66.36	44.72
Infant	100	131.04	46.30	29.36	19.56		
Ventilation mask	100	39.71	42.12	25.31	18.61		
Ventilation bag	41	1.42	4.26	1.28	2.89		
Nurse	59	0.59	1.10	0.74	1.93		
Others	97	6.67	9.71	9.67	14.34		
**AOI (Ventilation task)**						104.38	46.88
Infant	100	91.85	44.63	42.64	21.96		
Ventilation mask	100	70.76	41.78	40.18	14.28		
Ventilation bag	62	1.47	3.16	2.36	4.57		
Nurse	36	0.48	1.10	0.33	0.84		
Others	100	11.36	11.07	18.87	19.26		

*AOI, area of interest; CC, chest compression.*

Visual attention partly predicted the quality of CCs. Higher number of revisits to the area outside of the predefined AOIs negatively correlated with the total CC quality score (*r* = −0.35, *p* = 0.031; Table 2). Similarly, dwell time on the “outside” area negatively predicted the total CC quality score (*r* = −0.38, *p* = 0.018), whereas dwelling on infant chest positively correlated with percentages of correct CC rate (*r* = 0.33, *p* = 0.044; Table 3). The number of transitions between AOIs was unrelated to CC parameters. These results suggest that shifting gaze outside of the predefined AOIs, indicating distraction from the task at hand, may harm the quality of CCs, whereas focusing on the infant chest supports CC rate compliance.

**TABLE 2:** Correlations of revisits and transition count with chest compression and ventilation parameters.

	Revisits					Transitions
	Infant	Ventilation mask	Ventilation bag	Nurse	Others	
**Chest compression**						
Total CC score	−0.22	−0.02	0.04	0.04	−0.35*	−0.24
Correct hand position	−0.19	0.00	−0.05	0.04	−0.27	−0.22
Full recoil	0.09	0.16	0.05	−0.14	0.04	0.13
Mean CC depth	0.04	−0.04	−0.07	−0.08	−0.18	−0.06
CCs with correct depth	−0.12	−0.17	−0.06	0.04	−0.29	−0.22
Mean CC rate	0.10	0.01	−0.12	0.20	−0.07	0.03
CCs with correct rate	−0.09	−0.13	−0.01	−0.18	−0.17	−0.08
**Ventilation**						
Inspiratory tidal volume	0.07	0.41*	0.36*	−0.15	0.29	0.30
Expiratory tidal volume	0.12	−0.06	−0.26	−0.11	−0.04	−0.01
Peak inflation pressure	0.13	−0.03	−0.31	−0.04	−0.11	−0.03
Mask leak	0.15	0.50**	0.46**	−0.05	0.34*	0.40*

*p < 0.05, **p < 0.01. Significant correlations are marked in bold.

**TABLE 3:** Correlations of dwell time with chest compression and ventilation parameters.

	Dwell time				
	Infant	Ventilation mask	Ventilation bag	Nurse	Others
**Chest compression**					
Total CC score	0.26	−0.04	0.12	0.10	−0.38*
Correct hand position	0.21	−0.06	0.00	0.06	−0.31
Full recoil	0.05	0.05	0.06	−0.09	0.03
Mean CC depth	−0.04	0.01	0.04	−0.05	−0.09
CCs with correct depth	0.05	−0.03	0.06	0.06	−0.20
Mean CC rate	−0.14	0.01	−0.09	0.19	−0.02
CCs with correct rate	0.33*	−0.28	0.04	−0.22	−0.15
**Ventilation**					
Inspiratory tidal volume	−0.11	0.10	0.29	−0.08	0.13
Expiratory tidal volume	0.27	−0.25	−0.21	0.03	0.01
Peak inflation pressure	0.25	−0.17	−0.26	0.06	−0.01
Mask leak	−0.34*	0.27	0.38*	−0.15	0.17

*p < 0.05. Significant correlations are marked in bold.

### Visual Attention and Ventilation Quality

Similar to the CC scenario, the most salient AOIs in the ventilation scenario were infant chest and ventilation mask (both ratios = 100%; Table 1). The study nurse AOI had a ratio of 36% and the ventilation bag AOI had a ratio of 62%. There were significant differences in revisits among the AOIs (*F* = 98.60, *p* < 0.001), indicating that some AOIs were revisited more often than others (Figure 2). Again, revisits to the infant chest and ventilation mask AOIs were more frequent than revisits to any other AOI (*p* < 0.001). Participants (ratio = 100%) also focused on other stimuli than the predefined task-relevant AOIs and revisited the “others” AOI more often than the study nurse and ventilation bag AOIs (both *p* < 0.001).

High number of revisits to ventilation mask (*r* = 0.50, *p* = 0.002), ventilation bag (*r* = 0.46, *p* = 0.004), and the “others” AOI (*r* = 0.34, *p* = 0.042) correlated with high leakage, and revisits to ventilation mask (*r* = 0.41, *p* = 0.012) and ventilation bag (*r* = 0.36, *p* = 0.028) were also associated with higher inspiratory tidal volume (Table 2). Participants with V_*Ti*_ in the 4–8 mL/kg range revisited the ventilation mask and ventilation bag AOIs less frequently than participants with V_*Ti*_ out of that range (36.2 vs. 41.2 and 2.0 vs. 4.5 revisits for ventilation mask and ventilation bag, respectively). Moreover, the overall high number of transitions between AOIs correlated with high leakage (*r* = 0.40, *p* = 0.014). Regarding dwell time, dwelling on infant chest was associated with lower leakage (*r* = −0.34, *p* = 0.042), whereas dwelling on ventilation bag correlated with higher leakage (*r* = 0.38, *p* = 0.020; Table 3). Overall, these results indicate that participants who frequently switched their gaze between the ventilation equipment AOIs and other AOIs had troubles with the ventilation task at hand, whereas participants who dwelled longer on the infant chest dealt with the task better.

## Discussion

This study analyzed visual attention in a simulated pediatric resuscitation. We hypothesized that the infant and airway equipment would be the most salient cues for the resuscitation providers. In line with the hypothesis, we found that infant chest and ventilation mask (but not ventilation bag) were the most salient cues, as indicated by 100% ratio for both AOIs and the highest number of revisits to those AOIs. We further hypothesized that focusing on the infant and airway equipment would be related to CC and ventilation parameters. In a partial support of this hypothesis, we found that dwelling on the infant chest was associated with more correct CC rate in the chest compression task and lower leakage in the ventilation task, whereas dwelling on ventilation mask was unrelated to CC and ventilation parameters. Dwelling on ventilation bag and high number of revisits to airway equipment (both mask and bag) were even negatively related to mask leak during ventilations. Paying attention to the area outside of the predefined AOIs, indicating distraction from the task at hand, was overall negatively related to the total CC quality score.

Our findings indicate that the infant chest is the most important source of information for high CC rate compliance and low mask leak during pediatric resuscitation, which is congruent with prior evidence observed in pediatric intensive care unit consultants (16). This is in line with the current resuscitation guidelines that call for visual monitoring of adequate chest expansion during ventilation procedures (19). Clinically, though, this may prove difficult in children (21) and especially in preterm infants (22). Correct assessment of ventilation is essential to deliver sufficient but not excessive respiratory support. Feedback tools such as respiratory function monitors, end tidal CO_2_ measurements, or a coaching by observers may be profitably used to help in these situations (23, 24).

Even though high number of revisits typically indicates higher salience or importance of an AOI (20), we found that too many revisits to ventilation equipment and too frequent gaze transitions were associated with poor performance in the ventilation scenario (high mask leak, V_*Ti*_ out of optimal range). This was not expected and rather surprising. An explanation is that too frequent gaze shifts between the ventilation equipment AOIs and other AOIs were indicative of having troubles with the ventilation task. Participants might thus switch their gaze frequently to search for more information that would help them to better deal with the task at hand. Alternatively, the frequent gaze shifts might be indicative of insecurity and high nervousness which, in turn, negatively affected the ventilation quality. Our correlational design does not allow for drawing final conclusion for which of the above explanations is correct. Future research with experimental design is necessary to shed more light on the gaze shifting-performance causality.

However, the above suggestion about the relatively high numbers of revisits as a sign of nervousness or insecurity seems likely when considering results in the chest compression task. Contrasting the correlation between revisits to the infant chest and the total CC score with the correlation between dwell time on the infant AOI and the total CC score, we observed that the former correlation was negative and the latter positive (even though both non-significant). This indicates that frequent gaze shift away from the infant chest negatively correlated with overall CC performance, whereas dwelling longer on the infant chest without moving gaze back and forth too often (indicative of more stable, composed attention) predicted good performance. This also mirrors previously reported effects of stress on attentional resources and distractibility (25) and may be generalized to the simulation setting, as studies with simulated medical tasks typically produce acute stress responses in participants (26–28).

Notably, we found that paying attention outside of the predefined task-relevant AOIs (infant chest, ventilation mask, ventilation bag, and study nurse) correlated negatively with the total CC quality score and mask leak. The “others” AOI may be considered task-irrelevant and attention paid to this task-irrelevant AOI thus indicates distraction. Recent research has already shown that distracting healthcare providers either by external or internal stimuli during a resuscitation procedure resulted in lower quality of resuscitation (29–31). In those studies, distractors mainly operated as emotional stressors, with resuscitation circumstances being modified by the addition of noises, interference by actors, or cognitive tasks. In our study, however, no internal or external distractions were manipulated, yet many providers still diverted their attention from desired areas of focus, thereby diminishing the reception of information and harming their performance. The reasons for this distraction are unclear. It might be curiosity, insecurity, inexperience, feeling responsible for the overall management of the infant, and many others. Qualitative, semi-structured interviews could potentially help to better understand the underlying reasons for this distraction together with how to help providers stay focused on their task.

This study has both strengths and limitations. Strengths include objectively measured resuscitation performance, standardization, and a broad range of parameters tested. Moreover, the study extends prior research by directly testing the relationship between gaze behavior and resuscitation performance, which is a novel contribution given that recent researchers so far examined gaze differences of HCPs without directly relating the gaze to resuscitation performance (11–16). Limitations are the simulation-based character of the study not involving any real patients and the correlational design which does not allow for any causal interpretation of the findings. Our study design also did not allow to combine the components of resuscitation (CCs, ventilations) to give an overall estimate on pediatric resuscitation quality. Furthermore, our study included a rather small sample, which means that we cannot exclude the possibility of type-II errors (i.e., missed some important correlations). With a sample size of 40, however, the study was sensitive enough to detect correlations of 0.21 and higher. We tested revisits and dwell times on five different AOIs and related them to seven CC and four ventilation parameters. With this many tests we cannot exclude the possibility of a type-I statistical error. Indeed, if an adjustment was applied to the *p*-values for these correlations (e.g., Bonferroni, Holm-Bonferroni, and Chow-Denning), none would have been statistically significant. There is an ongoing debate about whether and when to use significance adjustments, with some researchers advocating the adjustments based on the results from computer modeling with random numbers (32), whereas other researchers recommending no adjustments because the data under evaluation are not random numbers but actual observations and adjusting *p*-values can potentially mask important findings (33). Given that significance adjustments are concerned with the general null hypothesis [i.e., that all null hypotheses are true simultaneously; (34)], it seems reasonable to use them for *post hoc* comparisons because the general null hypothesis has been already tested in the main model, yet the adjustments seem to be of little relevance for a sole assessment of individual relationships (e.g., in a correlation matrix). Still, due to small sample size and multiple tests, our results should be taken with caution and replicated with larger samples before final conclusions can be drawn. Finally, it would be interesting to measure effects of different levels of expertise, but there was a disproportional distribution of students, fellows, and consultants in our sample, which did not allow for meaningful comparisons. Learning more about the value and patterns of visual attention in experts remains an important issue for future research in order to improve current medical training for maximum benefits.

## Conclusion

We examined patterns of visual attention in providers during a simulated pediatric resuscitation and found that concentrating on the infant’s chest during both CC and ventilation tasks correlated with better CC rate compliance and lower mask leak. In contrast, focusing on task-irrelevant environment, indicating distraction, was related to poor outcomes. As recommended in the current pediatric resuscitation guidelines, trainers should teach providers to concentrate on patient chest for improved performance.

## Data Availability Statement

The raw data supporting the conclusions of this article will be made available by the authors, without undue reservation.

## Ethics Statement

The studies involving human participants were reviewed and approved by the Medical University of Vienna. The patients/participants provided their written informed consent to participate in this study.

## Author Contributions

PG, MW, and FC conceived and designed the study, and wrote the manuscript. FE, MW, PG, and FC performed the study. KB and HS helped with coordination of the study and coding eye-tracking data. PG and MW analyzed and interpreted the data. KB, HS, and AB critically reviewed and revised the manuscript for important intellectual content. All authors approved the final manuscript as submitted and agreed to be accountable for all aspects of the work.

## Conflict of Interest

The authors declare that the research was conducted in the absence of any commercial or financial relationships that could be construed as a potential conflict of interest.

## Publisher’s Note

All claims expressed in this article are solely those of the authors and do not necessarily represent those of their affiliated organizations, or those of the publisher, the editors and the reviewers. Any product that may be evaluated in this article, or claim that may be made by its manufacturer, is not guaranteed or endorsed by the publisher.
